# Pancreaticobiliary Maljunction and Its Relationship with Biliary Cancer: An Updated and Comprehensive Systematic Review and Meta-Analysis on Behalf of TROGSS—The Robotic Global Surgical Society

**DOI:** 10.3390/cancers17010122

**Published:** 2025-01-02

**Authors:** Yeisson Rivero-Moreno, Aman Goyal, Victor Bolívar, Nnenna Osagwu, Sophia Echevarria, José Gasca-Insuasti, Freddy Pereira-Graterol, Dagny von Ahrens, Omar Felipe Gaytán Fuentes, Luis Osvaldo Suárez-Carreón, Miljana Vladimirov, Beniamino Pascotto, Juan Santiago Azagra, Natale Calomino, Adel Abou-Mrad, Luigi Marano, Rodolfo J. Oviedo

**Affiliations:** 1 Montefiore Medical Center, Department of Surgery, New York, NY 10451, USA; yriveromor@montefiore.org (Y.R.-M.); dvonah@montefiore.org (D.v.A.); 2 Universidad de Oriente, Núcleo Anzoátegui, Anzoátegui 6001, Venezuela; 3 Adesh Institute of Medical Sciences and Research, Bathinda 151001, Punjab, India; doc.aman.goyal@gmail.com; 4 McGovern Medical School at UTHealth, Houston, TX 77002, USA; victor.bolivar@uth.tmc.edu; 5 School of Medicine, All Saints University, Roseau 5415, Dominica; nenna.udeh@allsaintsuniversity.org; 6 Universidad Mayor de San Simon, Cochabamba E-0352, Bolivia; sophia.echevarria@gmail.com; 7 Universidad Santiago de Cali, Cali 763531, Colombia; jose.gasca00@usc.edu.co; 8 Minimal Invasive Unit, “Dr. Luís Razetti” University Hospital, Barcelona 6001, Venezuela; freddypereiragraterol@gmail.com; 9 General Surgery Department, “CMN 20 de Noviembre” ISSSTE, México City 03104, Mexico; ganf29@hotmail.com; 10 Revenant Clinic, Integral Obesity Clinic—Hospital Ángeles Acoxpa, México City 14308, Mexico; 11 Bariatric Surgical Services, UMAE Hospital de Especialidades del Centro Medico Nacional de Occidente, Guadalajara 44329, Mexico; osvaldo.suarez@academico.udg.mx; 12 Universidad de Guadalajara, Guadalajara 44100, Mexico; 13 Department of General and Visceral Surgery, Bielefeld University, Campus Lippe, 33604 Bielefeld, Germany; miljana.vladimirov@klinikum-lippe.de; 14 Centre Hospitalier de Luxembourg, 1210 Luxembourg, Luxembourg; pascotto.beniamino@chl.lu (B.P.); azagra.js@chl.lu (J.S.A.); 15 Department of Medicine, Surgery, and Neurosciences, University of Siena, 53100 Siena, Italy; natale.calomino@unisi.it; 16 Department of Surgery, Centre Hospitalier Universitaire d’Orléans, 45100 Orléans, France; adel.abou-mrad@orange.fr; 17 Department of Medicine, Academy of Applied Medical and Social Sciences-AMiSNS, 82-300 Elblag, Poland; 18 Department of General Surgery and Surgical Oncology, “Saint Wojciech” Hospital, “Nicolaus Copernicus” Health Center, 80-462 Gdańsk, Poland; 19 Department of Surgery, Nacogdoches Medical Center, Nacogdoches, TX 75965, USA; 20 Department of Surgery, Tilman J. Fertitta Family College of Medicine, University of Houston, Houston, TX 77021, USA; 21 Department of Surgery, Sam Houston State University College of Osteopathic Medicine, Conroe, TX 77304, USA

**Keywords:** pancreatobiliary maljunction, biliary cancer, gallbladder, bile duct

## Abstract

Objective: This systematic review and meta-analysis aimed to determine the degree to which pancreaticobiliary maljunction (PBM) increases the risk of different types of biliary cancer (BC). Methods: A systematic review and meta-analysis were carried out using the following databases: PubMed, Embase, Cochrane Library, Scopus, Web of Science, and Science Direct. We systematically searched from inception to April 2024. The search terms included were derived from the keywords “Pancreaticobiliary Maljunction” OR “Anomalous Pancreaticobiliary Junction” AND “Cancer” OR “Malignancy”. Studies that provided data comparing BC rates in relation to PBM presence or vice versa were included. The Newcastle–Ottawa Scale (NOS) was used for quality assessment. The random-effects model was used. Results: Fifteen studies were included with a total sample of 8604 patients, of whom 5015 (58.29%) were female with a mean age of 54.58 years. Patients with PBM had 8.42 (95% CI = 3.57–19.87) more risk of developing any type of BC, with a higher risk of GBC than BDC (OR = 16.91 vs. OR = 3.36, *p*-value = 0.003). There was a higher risk of having PBM in patients with GBC than BDC only when considering the Asian population (OR = 3.12, 95% CI = 1.09–8.94). Meta-regression analysis revealed that neither mean age (*p* = 0.087) nor percentage of female patients in the study population (*p* = 0.197) were statistically associated with the variations in OR for the risk of BC based on the presence of PBM. Conclusions: There is a significant association between PBM and the risk of having BC, mainly GBC when compared to BDC. Most of the studies published reported data from Japanese patients, which limits the generalization of the results. The age of patients and sex were not significantly associated with the relation between PBM and BC. Further prospective studies in broader populations will provide additional details to take measures for screening and early management of PBM and BC.

## Simple Summary

This study explores the relationship between pancreaticobiliary maljunction (PBM) and the risk of developing biliary cancer (BC). PBM is a condition where the pancreatic and bile ducts join abnormally, and this research seeks to understand whether it increases the likelihood of certain cancers. By analyzing data from multiple studies, we found that individuals with PBM are significantly more likely to develop biliary cancers, especially gallbladder cancer (GBC), compared to bile duct cancer (BDC). The findings indicate that the risk may be elevated among Asian populations compared to other regions. Age and gender were not found to significantly influence this risk. These results emphasize the need for more widespread studies to help improve screening and early intervention strategies for those with PBM, potentially reducing cancer risks. The study highlights the importance of further research in diverse populations to better understand these associations.

## 1. Introduction

Pancreaticobiliary maljunction (PBM) was first recorded in the early 20th century and officially named in 1969 as a congenital defect of the pancreaticobiliary tract. The main anatomical feature of PBM is that the common bile duct and the pancreatic duct converge outside of the duodenal wall, forming a lengthy common duct. As a result, the pancreatic duct and the common bile duct exhibit decreased tone and control of pancreatic and biliary fluid flow, which causes reflux [1].

The incidence of PBM varies from 1.5% to 3.2%. The estimated prevalence in Asia is 100 to 1000 times higher than in other parts of the world, but some studies demonstrate pathological findings similar to those noted in the eastern population. Furthermore, it is a condition that is more common in females [2].

A major issue in patients with PBM is the risk of biliary cancer (BC) [3]. It is estimated that 10% of gallbladder cancer (GBC) cases are due to this anomaly [4]. Although it is a relatively rare biliary tract disorder, the risk that it carries for future biliary tract cancer makes it necessary to quantify it and identify the risk factors associated with its increase. This study aimed to determine the exact measure by which PBM could increase the risk of different types of BC through a systematic review and meta-analysis.

## 2. Materials and Methods

### 2.1. Study Design

A systematic review and meta-analysis were conducted to investigate the relationship between PBM and different types of BC. We registered the protocol of this systematic review in the International Prospective Register of Systematic Reviews (PROSPERO) under the number CRD42024532815. It was conducted according to the Preferred Reporting Items for Systematic Review and Meta-Analysis Protocols (PRISMA-P 2020) guidelines [5]. Approval from an Institutional Review Board (IRB) committee was not necessary or indicated, as we used data from published primary studies available in the literature.

This research methodology investigated the correlation between PBM and the risk of various types of BC in both adult and pediatric patients. It compared individuals with this condition to those with a normal structured biliary system to assess the likelihood of diagnosing any form of BC. The central question driving the study was to what extent PBM elevates the risk of different types of BC.

The definition of PBM considered for the study was the one presented by Kamisawa et al. and the Japanese Study Group on Pancreaticobiliary Maljunction (JSPBM) in 2013 as “a congenital malformation in which the pancreatic and bile ducts join anatomically outside the duodenal wall” [6]. All the studies presented used the same definition, although some utilized different classification systems for the diseases based on the types of confluence between the distal common bile duct as well as the morphology of the common channel, such as the one from Kimura Komi, and finally, the one presented by JSPBM in 2015 [7]. The classification system used in the different studies did not affect the analysis of the results, as variables were not included based on the specific type of PBM.

### 2.2. Information Sources and Search Strategy

A comprehensive search strategy was developed to search the following databases: PubMed, Embase, Cochrane Library, Scopus, Web of Science, and Science Direct. We systematically searched those databases from the first published studies in the literature to April 2024. The search terms included were derived from the keywords “Pancreaticobiliary Maljunction” OR “Anomalous Pancreaticobiliary Junction” AND “Cancer” OR “Malignancy”. The search was conducted on 1 April 2024. A complete list of search terms and a detailed search strategy are included in Supplementary Table S1.

### 2.3. Selection Process

Two independent reviewers evaluated the titles and abstracts of identified studies to determine their relevance based on the inclusion and exclusion criteria. The full text of studies deemed potentially relevant were subsequently reviewed for final eligibility. Any discrepancies between the reviewers were resolved through discussion or, if necessary, consultation with a third independent reviewer. The inclusion criteria encompassed studies that provide data on the frequencies of BC and PBM as well as those that compare BC rates in relation to PBM presence or vice versa. Eligible study designs include cohort, case-control, and cross-sectional studies. The exclusion criteria involved case reports, case series, reviews, commentaries, conference abstracts, and editorials, along with studies featuring overlapping populations or not conducted in English. Notably, there were no restrictions based on sample size, patient age, or publication timeframe.

### 2.4. Data Extraction

Reviewers extracted data from eligible studies using a standardized form in an Excel template. The extracted data included study characteristics (year of publication, country of origin, sample size, study design, and year of publication), patient characteristics (age, sex, and classification used for PBM), type of cancer registered, and the presence of PBM.

### 2.5. Bias and Quality Assessment

The Newcastle–Ottawa Scale (NOS) was used for the evaluation of observational studies by two independent reviewers to assess the quality of the included studies. The total score can range from 0 to 9 stars. Studies with a score of 7–9 were graded as high quality, 4–6 as medium quality, and a score of <4 as poor quality [8]. Any discrepancies between reviewers were resolved through discussion or consultation with a third independent reviewer.

### 2.6. Statistical Analysis

Exposure effects for binary endpoints were compared using pooled odds ratios (OR) with 95% confidence intervals (CI); absolute risk differences (RD) were also presented with 95% CI. The findings were displayed in tables delineating the primary study and patient characteristics, along with forest plots illustrating the aggregated results. We assessed heterogeneity for each outcome using I^2^ and defined I^2^ of 75% and greater as substantial heterogeneity, 25–75% as moderate heterogeneity, and below 25% as low heterogeneity, as defined by Higgins et al. [9]; *p* values inferior to 0.10 and I² > 25% were considered significant for heterogeneity. In pooled outcomes with high heterogeneity, the DerSimonian and Laird random-effects model was used [10]. Review Manager 5.4 (Nordic Cochrane Centre, The Cochrane Collaboration, Copenhagen, Denmark) and SPSS V.29 were used for statistical analysis.

In order to evaluate potential confounders in the relation between the prevalence of BC and PBM, we performed a subgroup analysis based on the specific type of cancer: gallbladder cancer (GBC) or bile duct cancer (BDC). A sensitivity analysis was also performed to ensure the results were not dependent on a specific type of study. Furthermore, a univariate meta-regression was conducted to further explore potential sources of heterogeneity, including mean age and percentage of females.

Publication bias was evaluated by visual inspection of funnel-plot graphs to assess for the symmetrical distribution of trials with similar weights and Egger’s test. An asymmetrical funnel plot and a *p*-value < 0.1 on Egger’s test indicated the presence of publication bias [11].

## 3. Results

### 3.1. Study Selection

**Figure 1:**
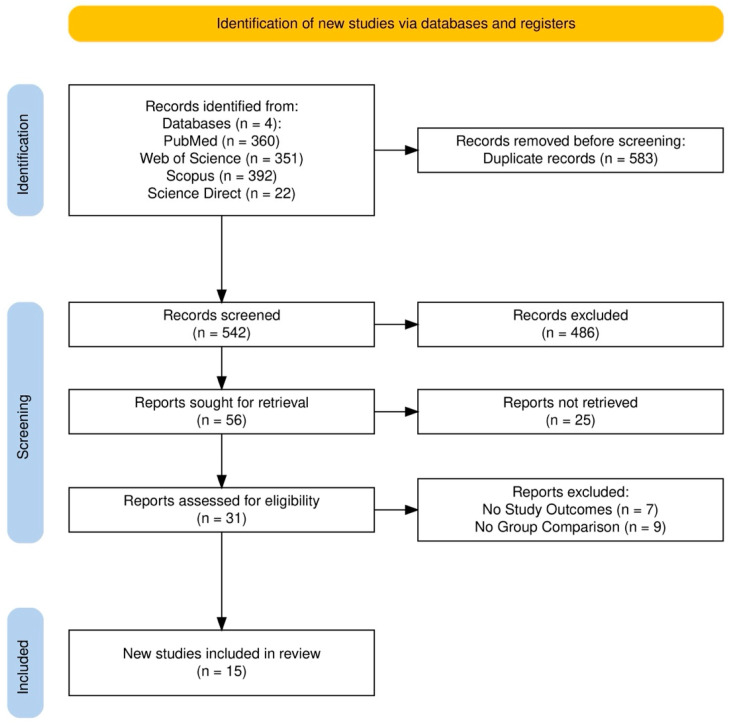
Search outputs based on PRISMA guidelines [13].

An initial search across the four databases yielded 1125 records, of which 583 were duplicates. After removing the duplicates, 542 unique records underwent title and abstract screening, leading to the selection of 56 studies for full-text review based on predetermined inclusion and exclusion criteria. Of these, 25 studies were excluded due to unavailability. Following the full-text review, data were finally extracted data from 15 articles, all written in English. The reasons for exclusion and the study selection process are illustrated in Figure 1, which follows the PRIMSA guidelines and was structured through the Haddaway et al. online app [12].

### 3.2. Study Characteristics

The 15 included studies were published between 1997 and 2019, with a median year of 2006. Most of them were cross-sectional studies (*n* = 13, 80%) and three cohort studies. Japan was the most common country of origin of the patients evaluated with a total of 10 studies (66.7%), followed by China with two studies (13.3%) and the rest from Greece, the USA, South Korea, and Taiwan, with one study each (6.6%). Six studies (40%) reported having used JSPBM to define and further classify the cases of PBM [14]. Nine studies (60%) reported data specifically separated for the bile duct and GBC. The detailed features of the included studies and description of the population are shown in Table 1. After applying the inclusion criteria, since only one study was from outside Asia, we decided to continue the analysis based solely on the 14 Asian studies.

### 3.3. Results of Syntheses

The total sample comprised 8546 patients. Among them, 4983 were female (58.3%) with a mean age of 54.58 years. Overall, seven studies reported the incidence of BC based on the presence of PBM, six reported the presence of PBM based on the type of BC (bile duct vs. gallbladder), and one reported the presence of PBM based on the presence of GBC.

### 3.4. Risk of BC in Patients Based on the Presence of PBM

**Figure 2:**
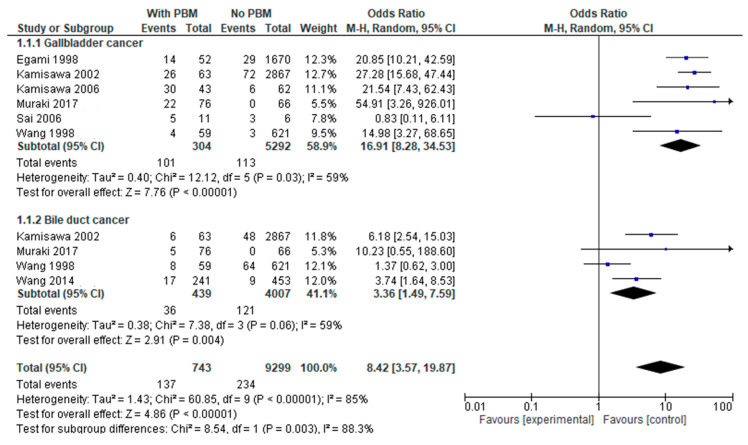
Forest plot of the risk of BC in patients based on the presence of PBM.

We evaluated a pooled OR for BC among 6290 patients based on the presence (n = 545, 8.7%) or absence (n = 5745, 91.3%) of PBM, resulting in patients with PBM having 8.42 (95% CI = 3.57–19.87) more risk of developing any type of BC (Figure 2). When analyzed by subgroups of type of BC, there was a higher risk of developing GBC than BDC (OR = 16.91 vs. OR = 3.36, *p*-value = 0.003) in patients with PBM, with a statistically significant difference and an RD of 0.28 for GBC vs. 0.06 for BDC.

### 3.5. Risk of PBM in Patients Based on the Type of BC

We evaluated a pooled OR for the frequency of PBM among 1176 patients based on the presence of BDC (n = 614, 52.2%) or GBC (n = 562, 47.8%). There was a higher OR to have PBM in patients with GBC. However, it was not statistically significant, OR = 1.98 (95% CI = 0.58–6.76); but a different result was found in the sensitivity analysis shown later. The RD for this outcome was 0.04 (95% CI = −0.07–0.16).

### 3.6. Risk of PBM Based on the Presence of BC

**Figure 3:**
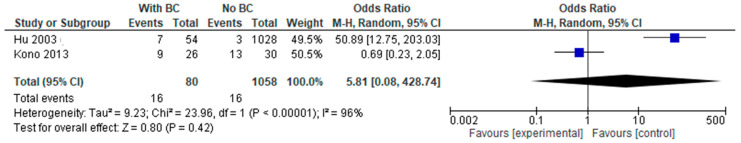
Forest plot of the risk of PBM based on the presence of BC.

Furthermore, we evaluated a pooled OR for the frequency of PBM among 1138 patients based on the presence (n = 80, 7%) or absence (n = 1058, 93%) of GBC. There was a higher OR to have PBM in patients with GBC. However, it was not statistically significant, OR = 5.81 (95% CI = 0.08–428.74) (Figure 3). The RD for this outcome was 0.05 (95% CI = 0.16–0.26).

### 3.7. Sensitivity Analysis and Meta-Regression

We performed a sensitivity analysis withdrawing studies from Western populations, like the Roukounakis et al. study in Greece [24], from the pooled analysis to address differences in the frequency of PBM in patients with GBC vs. those with BDC. It was also observed that in the Asian population (Japan and South Korea), patients with GBC had a higher prevalence of PBM compared to patients with BDC (OR = 3.12, 95% CI = 1.09–8.94).

We also removed studies with sample sizes of less than 50 patients [25]. The results of this sensitivity analysis were consistent with the primary analysis for the risk of GBC in patients with PBM but increased the risk considerably (OR = 23.81, 95% CI = 16.16–35.07).

Prespecified meta-regression was performed, revealing that the mean age (*p* = 0.087) and percentage of female patients in the study population (*p* = 0.197) were not statistically associated with the variations in OR for the risk of BC based on the presence of PBM. Details are provided in Figures S1 and S2 of the Supplementary Materials.

### 3.8. Heterogeneity and Risk of Bias Across Studies

There was moderate to substantial heterogeneity in all the outcomes analyzed (risk of BC based on the presence of PBM: I^2^ = 85%; risk of PBM based on type of BC: I^2^ = 84%; and risk of PBM based on the presence of BC: I^2^ = 96%).

**Figure 4:**
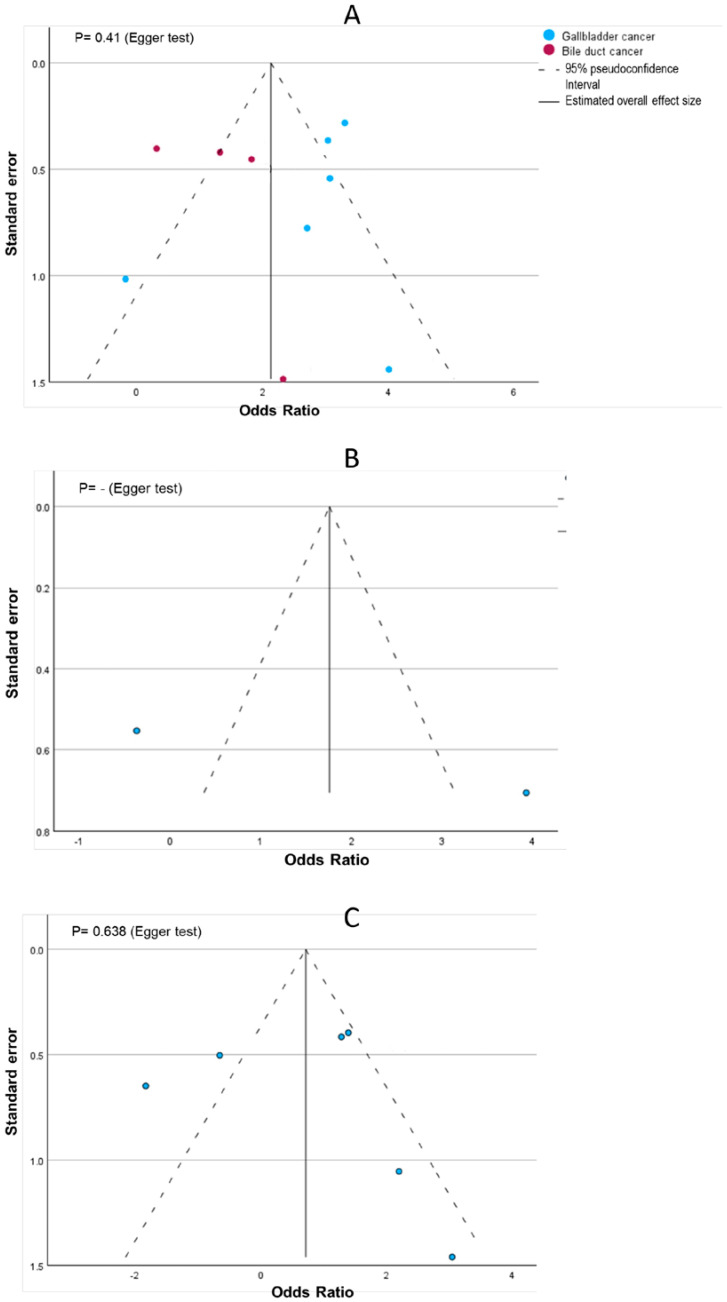
Funnel plots of the studied outcomes (the solid line represents the estimated overall effect size, and the dashed line is the 95% pseudoconfidence interval. (**A**): risk of BC based on the presence of PBM; (**B**): risk of PBM based on type of BC; (**C**): risk of PBM based on the presence of BC) [15,16,17,18,19,20,21,22,23,24,25,26,27,28,29].

The comparison-adjusted funnel plots and Egger’s test for the different outcomes are shown in Figure 4. The Egger test for studies evaluating the risk of BC based on the presence of PBM yielded a result of *p* < 0.1, suggesting publication bias. However, none of Egger’s tests for the other outcomes yielded a result of *p* < 0.1, suggesting the absence of publication bias in those included studies.

### 3.9. Quality Assessment

The Newcastle–Ottawa scale is presented in Table S2 of the Supplementary Materials. The majority of studies received scores ranging from 7 to 9, with a median score of 8.1, indicating high quality.

## 4. Discussion

The primary aim of this study was to determine the association between PBM and the risk of developing BC, specifically GBC and BDC. Our systematic review and meta-analysis revealed a significant association between PBM and increased risk of BC. According to the GLOBOCAN 2022, the incidence of GBC is 0.6%, with 122,462 new cases annually and a total mortality rate of 0.9%, accounting for 89,031 deaths [30]. Notably, Chile has one of the highest GBC mortality rates globally [31]. Across 15 studies spanning from 1997 to 2019, predominantly focusing on Japanese populations, we observed that patients with PBM had higher odds of developing BC compared to those without PBM. Within the subset of BC patients, those with PBM showed a significantly elevated risk of GBC (OR = 16.91) and BDC (OR = 3.36).

PBM is a congenital anomaly characterized by the anatomical joining of the pancreatic and bile ducts outside the duodenal wall. This malformation results in the duodenal papillary sphincter (sphincter of Oddi) being unable to regulate the pancreaticobiliary junction due to an abnormally long common channel. Consequently, reflux of pancreatic juice and bile occurs, leading to a range of pathological conditions, including impaired excretion of bile and pancreatic juice and an increased risk of biliary cancer within the biliary tract and pancreas [32,33].

Our findings were consistent with previous research, like one by Hyvärinen et al. (2019), who conducted a retrospective study on 225 patients using medical records and magnetic resonance cholangiopancreatography (MRCP) images from individuals treated for biliary malignancies between 2005 and 2016 [34]. Among those patients, PBM was identified in four individuals (5.5%) with interpretable MRCP. Out of these four patients, three suffered from GBC and one from extra-hepatic BDC. These results underscore the heightened risk of GBC associated with PBM, aligning with our meta-analysis findings [34].

Li Y et al. (2013) investigated the PBM association, specifically with common BDC, and analyzed 10 studies (8 case-control and 2 cohort). This meta-analysis also resulted in a positive association of PBM with common BDC (OR = 1.45, 95% CI: 1.19–1.76) [35].

Furthermore, a study by Deng YL et al. (2011) on the relationship between PBM and GBC also highlights this association. Deng YL’s meta-analysis, which included nine case-control studies, found that the incidence of PBM was higher in GBC patients than in controls (10.60% vs. 1.76%, OR: 7.41, 95% CI: 5.03 to 10.87). The study noted that female patients with PBM had a 1.96-fold higher proportion compared to GBC patients without PBM (80.5% vs. 62.9%, OR: 1.96, 95% CI: 1.09 to 3.52) [36].

PBM is predominantly common among women, indicating a significant gender-based disparity. Most patients identified in our study with PBM were female (58.29%; with a mean age of 54.58 years). This is consistent with the prior literature demonstrating a higher incidence of GBC development in women affected by PBM compared to their male counterparts [37]. A study reported by Hyvärinen et al. [34] revealed that PBM patients were significantly more likely to be female (100% vs. 43%) and exhibited a higher incidence of GBC (75% vs. 22%, *p* = 0.044) compared to patients without PBM.

This gender bias may stem from various influences, including the presence of gallstones, hormonal dynamics, and genetic predispositions [37]. However, it is crucial to delve deeper into the exploration of additional causal factors, including environmental influences, particularly considering the higher prevalence of cases in Asia. Further research in this realm is warranted to comprehensively understand the multifactorial nature of PBM incidence.

In our review, we included 15 studies, of which 10 (66.7%) were from Japan. The higher incidence of PBM in Japan can be attributed to a combination of genetic, environmental, and healthcare factors. Genetic predispositions and specific dietary habits or environmental exposures may contribute to this condition. Japan’s advanced healthcare system, with regular health screenings and the use of sophisticated diagnostic techniques, results in higher detection rates of PBM [38]. Additionally, a strong research focus on biliary and pancreatic diseases in Japan leads to more frequent reporting of PBM cases. Cultural and epidemiological factors, such as the prevalence of related conditions like gallstones, also play a role, further compounded by potential reporting biases due to extensive medical research activities in the country [38].

While the JSPBM definition is widely recognized in the literature and was used by six studies included in our analysis, it is important to note that the remaining studies did not provide specific details on the classification criteria or only reporting the presence or absence of PBM without further elaboration on the subtypes. Despite these variations, the absence of detailed subtype-related variables in our analysis minimizes the potential impact of these differences on the overall findings.

The predominantly observational nature of the included studies introduces inherent biases and confounding factors. Additionally, variations in study methodologies, diagnostic criteria, follow-up durations, and patient populations contribute to the high heterogeneity observed in our analysis. Moreover, the absence of consistent data on bile duct dilation and other common bile duct malformations across studies precluded a more detailed subgroup analysis, which could potentially refine our understanding of their role in biliary carcinogenesis. The studies included in the analysis were cross-sectional rather than longitudinal studies, which limits the causality and long-term outcomes. Furthermore, many of the studies analyzed in this review are older, with publication dates ranging from 1997 to 2019 and a mean publication year of approximately 2006.

Although efforts were made to include all of the available studies, the absence of recent original research underscores the need for updated investigations. Additionally, there are relatively few studies from regions outside of Asia, limiting the generalizability of our findings to other parts of the world. In order to keep the analysis more homogeneous, we have excluded the non-Asian population [23,24] from the sensitivity analysis to address differences in the frequency of PBM in patients with GBC versus those with BDC. This approach was aimed at mitigating potential regional disparities and enhancing the consistency of the findings across studies.

Despite these limitations, our research provides valuable insights into the complex interplay between PBM and the risk of different types of BC in a detailed manner. Prospective studies incorporating larger more diverse patient populations, standardized diagnostic criteria, and mechanistic investigations into the pathophysiology of biliary carcinogenesis are warranted. Additionally, exploring the efficacy of targeted interventions, such as early screening, surgery, and immunomodulatory therapies, holds promise in mitigating the burden of BC in high-risk populations.

## 5. Conclusions

There is a significant association between PBM and the risk of having BC, especially GBC vs. BDC. Most of the studies published reported data from Japanese patients, which limits the generalization of the results. The age of patients and sex were not significantly associated with the relationship between PBM and BC, although most cases with PBM are female patients.

## Figures and Tables

**Table 1:** Characteristics of the included studies.

Author	Year Published	Country	Type of Study	Sample Size	Age Mean	Female (%)	PBM Classification Used	Type of Cancer
Cho et al. [15]	2011	South Korea	Cross-sectional	20	40.2	75	NR	BDC and GBC
Egami et al. [16]	1998	Japan	Cross-sectional	1722	55.4	NR	NR	GBC
Hu et al. [17]	2003	China	Cross-sectional	1082	59	NR	JSPBM	GBC
Kamisawa et al. [18]	2006	Japan	Cross-sectional	105	NR	NR	NR	GBC
Kamisawa et al. [19]	2010	Japan	Cohort	446	60.3	69.95	NR	BDC and GBC
Kamisawa et al. [20]	2002	Japan	Cross-sectional	2930	52.8	48.94	NR	BDC and GBC
Kono et al. [21]	2013	Japan	Cohort	56	65	67.85	NR	GBC
Lee et al. [22]	2011	Japan	Cohort	75	42	78.75	JSPBM	BDC and GBC
Muraki et al. [23]	2017	Japa	Cross-sectional	142	52.7	53.52	NR	BDC and GBC
Roukounakis et al. [24]	2006	Greece	Cross-sectional	58	66.5	55.17	NR	BDC and GBC
Sai et al. [25]	2006	Japan	Cross-sectional	17	54.1	52.94	NR	GBC
Sandoh et al. [26]	1997	Japan	Cross-sectional	106	56	NR	JSPBM	BDC and GBC
Takayashiki et al. [27]	2019	Japan	Cross-sectional	471	52	37.15	JSPBM	BDC and GBC
Wang et al. [28]	2014	China	Cross-sectional	694	51.8	43.37	JSPBM	BDC
Wang et al. [29]	1998	Taiwan	Cross-sectional	680	NR	NR	JSPBM	BDC and GBC

NR: not reported, JSPBM: Japanese Study Group Classification of Pancreaticobiliary Maljunction, PBM: Pancreaticobiliary Maljunction, GBC: Gallbladder Cancer, BDC: Bile duct cancer.

## Data Availability

The datasets generated and/or analyzed during this study are not publicly available but may be obtained from the corresponding author upon reasonable request.

## Supplementary Materials

The following supporting information can be downloaded at: https://www.mdpi.com/article/10.3390/cancers17010122/s1, Figure S1: Meta regression assessing the impact of the percentage of female in the study population on the LogOR for BC based on the presence of PBM; Figure S2: Meta regression assessing the impact of age on the LogOR for BC based on the presence of PBM; Table S1: Search terms for the meta-analysis in PubMed, Scopus, Web of Science databases, and Science Direct.
