# Supplementary material for: Pancreaticobiliary Maljunction and Its Relationship with Biliary Cancer: An Updated and Comprehensive Systematic Review and Meta-Analysis on Behalf of TROGSS—The Robotic Global Surgical Society
Source: Cancers (Basel). 2025 Jan 2;17(1):122. doi: 10.3390/cancers17010122 (PMC11719653; doi:10.3390/cancers17010122)
Supplement: Supplementary file 1 [file cancers-17-00122-s001.zip › cancers-3297273-supplementary.pdf]

Supplementary appendix

**Table S1:** Search terms for the meta-analysis in PubMed, Scopus, Web of Science databases, and Science Direct	
**Database**	**Search terms**
PubMed (n=360)	("Neoplasms"[Mesh] OR "Tumor"[Title/Abstract] OR "Neoplasm"[Title/Abstract] OR "Tumors"[Title/Abstract] OR "Neoplasia"[Title/Abstract] OR "Neoplasias"[Title/Abstract] OR "Cancer"[Title/Abstract] OR "Cancers"[Title/Abstract] OR "Malignant Neoplasm"[Title/Abstract] OR "Malignancy"[Title/Abstract] OR "Malignancies"[Title/Abstract] OR "Malignant Neoplasms"[Title/Abstract] OR "Malignant Neoplasm"[Title/Abstract]) AND ("Pancreaticobiliary Maljunction"[Mesh] OR "Pancreaticobiliary Maljunctions"[Title/Abstract] OR "Pancreaticobiliary Maljunction"[Title/Abstract] OR "Anomalous Pancreaticobiliary Junction"[Title/Abstract] OR "Anomalous Pancreaticobiliary Junctions"[Title/Abstract] OR "Anomalous Pancreaticobiliary Ductal Union"[Title/Abstract] OR "Anomalous Pancreaticobiliary Ductal Unions"[Title/Abstract] OR "Biliopancreatic Maljunctions"[Title/Abstract] OR "Biliopancreatic Maljunction"[Title/Abstract] OR "Anomalous Biliopancreatic Junction"[Title/Abstract] OR "Anomalous Biliopancreatic Junctions"[Title/Abstract] OR "Anomalous Biliopancreatic Ductal Union"[Title/Abstract] OR "Anomalous Biliopancreatic Ductal Unions"[Title/Abstract] OR "Anomalía de la unión biliopancreática"[Title/Abstract] OR "Anomalía de la unión pancreatobiliar"[Title/Abstract] OR "Unión biliopancreática anómala"[Title/Abstract] OR "unión pancreatobiliar anómala"[Title/Abstract] OR "mal unión pancreatobiliar"[Title/Abstract] OR "mal unión biliopancreática"[Title/Abstract])
Web of Science (n=351)	TS=(Tumor OR Neoplasm OR Tumors OR Neoplasia OR Neoplasias OR Cancer OR Cancers OR Malignant Neoplasm OR Malignancy OR Malignancies OR Malignant Neoplasms OR Malignant Neoplasm) AND TS=(Pancreaticobiliary Maljunctions OR Pancreaticobiliary Maljunction OR Anomalous Pancreaticobiliary Junction OR Anomalous Pancreaticobiliary Junctions OR Anomalous Pancreaticobiliary Ductal Union OR Anomalous Pancreaticobiliary Ductal Unions OR Biliopancreatic Maljunctions OR Biliopancreatic Maljunction OR Anomalous Biliopancreatic Junction OR Anomalous Biliopancreatic Junctions OR Anomalous Biliopancreatic Ductal Union OR Anomalous Biliopancreatic Ductal Unions OR Anomalía de la unión biliopancreática OR Anomalía de la unión pancreatobiliar OR Unión biliopancreática anómala OR unión pancreatobiliar anómala OR mal unión pancreatobiliar OR mal unión biliopancreática)
Scopus (n=392)	TITLE-ABS-KEY ("Tumor" OR "Neoplasm" OR "Tumors" OR "Neoplasia" OR "Neoplasias" OR "Cancer" OR "Cancers" OR "Malignant Neoplasm" OR "Malignancy" OR "Malignancies" OR "Malignant Neoplasms" OR "Malignant Neoplasm") AND TITLE-ABS-

	KEY ("Pancreaticobiliary Maljunctions" OR "Pancreaticobiliary Maljunction" OR "Anomalous Pancreaticobiliary Junction" OR "Anomalous Pancreaticobiliary Junctions" OR "Anomalous Pancreaticobiliary Ductal Union" OR "Anomalous Pancreaticobiliary Ductal Unions" OR "Biliopancreatic Maljunctions" OR "Biliopancreatic Maljunction" OR "Anomalous Biliopancreatic Junction" OR "Anomalous Biliopancreatic Junctions" OR "Anomalous Biliopancreatic Ductal Union" OR "Anomalous Biliopancreatic Ductal Unions" OR "Anomalía de la unión biliopancreática" OR "Anomalía de la unión pancreatobiliar" OR "Unión biliopancreática anómala" OR "unión pancreatobiliar anómala" OR "mal unión pancreatobiliar" OR "mal unión biliopancreática")
Science Direct (n=22)	Title, abstract, keywords: (cancer OR Neoplasm OR tumor) AND (Pancreaticobiliary Maljunction)"

Figure S1 - Meta regression assessing the impact of the percentage of female in the study population on the LogOR for BC based on the presence of PBM

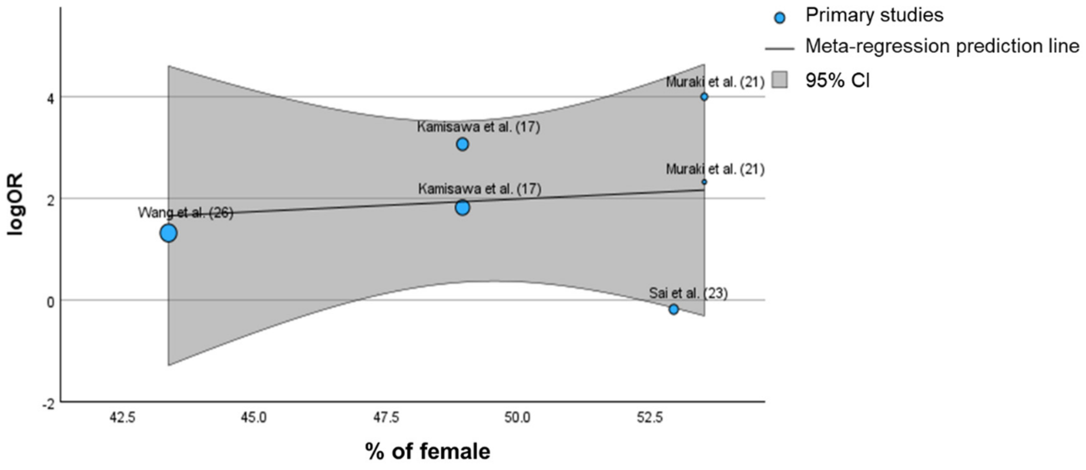

Variable	Effect Estimate	SE	p-value	Test for Residual Heterogeneity	I^2	R^2
% Female	0.090	0.238	0.726	P=0.20	70.8%	0%

Figure S2 - Meta regression assessing the impact of age on the LogOR for BC based on the presence of PBM

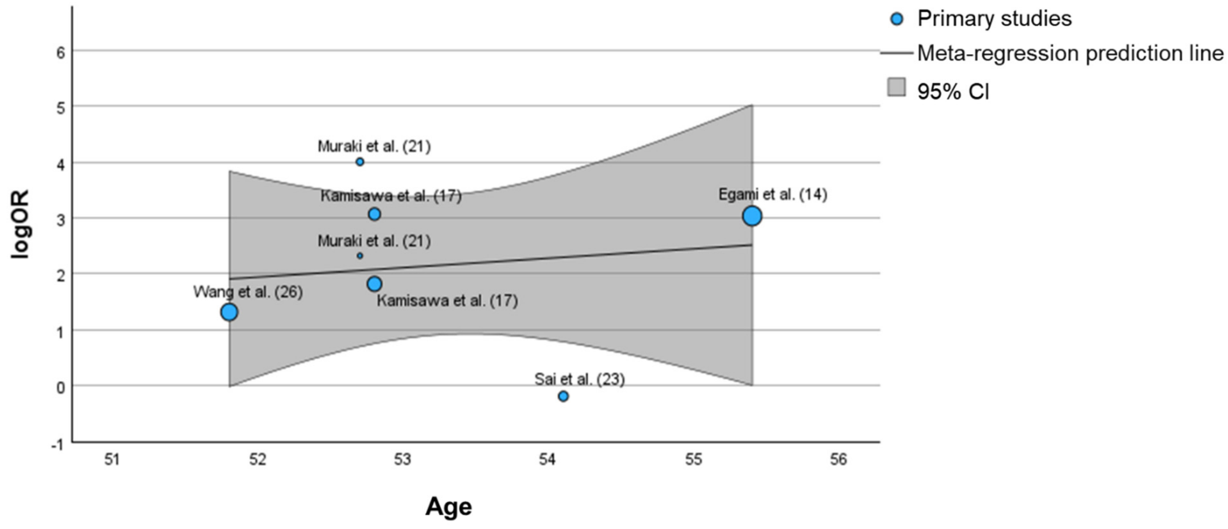

Variable	Effect Estimate	SE	p-value	Test for Residual Heterogeneity	I^2	R^2
Age	-0.578	0.693	0.442	P=0.19	70.7%	0%
